# Lidocaine for Reduction of Pain Induced by Propofol in a Tertiary Care Hospital: A Descriptive Cross-sectional Study

**DOI:** 10.31729/jnma.5796

**Published:** 2021-04-30

**Authors:** Binam Ghimire, Man Bahadur Chand

**Affiliations:** 1 Department of Anaesthesiology, Patan Academy of Health Sciences, Lalitpur, Nepal

**Keywords:** *lidocaine*, *pain*, *propofol*

## Abstract

**Introduction:**

Propofol is the most frequently used anaesthetic agent. Despite various anaesthetic benefits, propofol is not without side effects, pain on injection being the most common adverse effect. This study aimed to find the grade of pain reduced due to the injection of propofol after administration of lidocaine.

**Methods:**

A descriptive cross-sectional study was conducted from March 2015 to August 2015 in the operating theatre in a tertiary care hospital after taking ethical clearance with an ethical clearance from the Institutional Review Committee. A total of 64 participants fulfilling all inclusion criteria of both gender, age ranged from 16-65 years of American Society of Anesthesiologists physical status I and II ready for elective surgery under general anaesthesia with propofol pretreated with 60mg lidocaine with venous occlusion for one minute were observed. The pain was graded by the four-point scale (0=none, 1=mild, 2=moderate, 3=severe). Haemodynamic variables were measured until just before intubation.

**Results:**

In patients pretreated with lidocaine, no pain 56 (87.5%), mild pain 8 (12.5%) and moderate pain 0 (0%) were observed.

**Conclusions:**

The grade of pain during injection of propofol was reduced in more than three-fourth of the patients after administration of pre-anaesthetic drug-like lidocaine.

## INTRODUCTION

The introduction of general anaesthetic agents into clinical practice over 150 years ago stands as one of the seminal innovations of medicine. Propofol is the most frequently used anaesthetic today. Despite its various anaesthetic advantages, propofol is not without side effects; among which, pain on injection is the most commonly reported adverse effect.^1^

Several methods have been used to reduce this pain. Of all, lidocaine pretreatment with venous occlusion or injection in the large vein is most effective.^2,3^

Thus, the study aims to find the grade of pain reduced due to the injection of propofol after administration of lidocaine.

## METHODS

A descriptive cross-sectional study was conducted in the operating theatre at the National Academy of Medical Sciences (NAMS), Bir Hospital. Data collection was carried out for a six-month duration (from March 2015 to August 2015). Before commencing this study, ethical clearance with an ethical approval number of 134/14 was obtained from the Institutional Review Committee (IRC), NAMS, Bir Hospital.

Inclusion criteria were those who gave consent, patients belonging to the American Society of Anaesthesiologists (ASA) physical status I and II, and requiring elective surgery under general anaesthesia, the participants of both gender aged 16 to 65 years. Exclusion criteria were those unwilling to voluntarily participate in the study. Patients were also excluded if they had any known allergy to study drugs, had difficulty communicating, and if on concomitant use of pain modifying drugs like analgesic or sedative drugs within the 24 hours before surgery or had peripheral vascular diseases.

Convenience sampling method was used. Sample size was calculated using the formula:

$$\begin{array}{ll} \text{n} & \text{= }{\text{Z}}^{\text{2}}\times \text{p}\times \text{q}/{\text{e}}^{\text{2}}\\ & \text{= }{\left(1.96\right)}^{\text{2}}\times (0.5)(0.5)/{(\text{0.13)}}^{\text{2}}\\ & \text{= }57 \end{array}$$

where,

n = minimum sample size
Z = 1.96 at 95 % Confidence Interval
p = 50%, prevalence for maximum sample size
q = 1-p
e = margin of error, 13%

Taking 10% non-response rate of study participants. The sample size calculated was 63. However, the total sample taken was 64.

A total of 64 participants ready for elective surgery under general anaesthesia pretreated with 60mg lidocaine (volume of 5ml) with venous occlusion for one minute were observed. The pain was assessed by a four-point scale (0=none, 1=mild, 2=moderate, 3=severe). Haemodynamic variables were measured until just before intubation.

Written informed consent was taken from each participant of the study. A semi-structured questionnaire and an observational checklist were used for data collection and face to face interviews were taken. For the data collection, the pre-structured questionnaire was made by reviewing different literature and seeking opinions from the experts on the subject; thereafter appropriate modification was done. All patients fulfilling the inclusion criteria were noted in the questionnaire from the relevant patient file record. Data was collected before operation by face-to-face interview at the bedside along with a record file. Participation was voluntary and utmost confidentiality and personal identity of all the participants were assured.

After fully explaining all the procedures to the patients, venous occlusion was done by applying a venous tourniquet over mid-arm. The patients were injected intravenously 60mg preservative-free lidocaine diluted in 2ml of isotonic saline. Five ml disposable syringe was used. After 1 minute, the tourniquet was released followed by an injection of 25% of the total induction dose of propofol (2mg/kg) at a rate of 0.5ml/sec by an anaesthetic assistant. Patients were then assessed about pain according to four-point verbal categorical systems and behavioural signs as given below:

**Table:** Mc Crirrick and Hunter pain intensity scale.^4^

Pain score	Grade of pain	Response
0	None	The negative response to questioning
1	Mild	The pain reported in response to questioning only without any behavioural signs
2	Moderate	The pain reported in response to questioning and accompanied by behavioural signs (facial grimacing or withdrawal of hand or tears), or pain reported spontaneously without questioning
3	Severe	Strong verbal response, or response accompanied by behavioural signs (facial grimacing or withdrawal of hand or tears)

Hemodynamic parameters-heart rate and blood pressure were recorded two minutes before tourniquet as a baseline, after a test dose of propofol, after induction, before intubation. The collected data was entered in Microsoft Excel and converted into SPSS (Statistical Package for Social Science) software package 20 version for statistical analysis. Data were analyzed using descriptive statistics. Continuous data were expressed as mean and standard deviation and categorical variables as number and percentage.

## RESULTS

A total of 64 participants were enrolled in the study. There was no experience of severe and moderate pain by participants pretreated with lidocaine whereas mild pain was experienced by 8 (12.5%) participants. A higher no of participants that is 56 (87.5%) had no experience of any pain. The grade of pain on injection of propofol among lidocaine by verbal rating score after giving 25% of the calculated dose of propofol is shown (Table 1).

**Table 1:** Grade of pain on propofol injection in lidocaine.

Preanaesthetic agent	No Pain (Score 0) n (%)	Mild Pain (Score 1) n (%)	Moderate Pain (Score 2) n (%)	Severe Pain (Score 3) n (%)
Lidocaine (n= 64)	56 (87.5)	8 (12.5)	-	-

The baseline mean heart rates of 89.5±12.65 bpm in patients pretreated with lidocaine (Table 2).

**Table 2:** The pattern of mean heart rate among patients pretreated with lidocaine

Pre-anesthetic agent (lidocaine)	Mean heart rate
(n= 64)	89.5±12.65

The baseline mean arterial blood pressure among patients who are given lidocaine is 97.5±11.43 mm Hg. (Table 3).

**Table 3:** The pattern of mean arterial blood pressures (MAP) among patients pretreated with lidocaine.

Pre-anesthetic agent (lidocaine)	Mean arterial blood pressures (MAP)
(n= 64)	97.5±11.43

**Figure 1.**
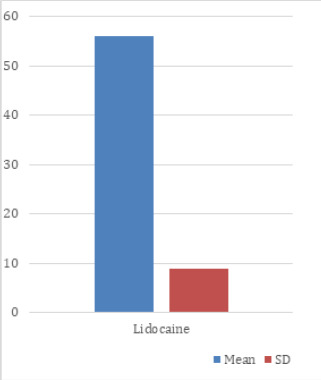
Mean age of the patients who are given lidocaine.

The minimum and maximum age of the patients were 16 and 60 years in patients pretreated with lidocaine. Likewise, the mean age of the patients were 37.69±13.34 years to whom lidocaine was given (Figure 1).

The mean weight of the patients was 56.11±8.82 kg in patients who were given lidocaine. Similarly, out of the total number of 64 patients pretreated lidocaine, 53 (83.3%) were of ASA I and 11 (16.7%) were of ASA II.

## DISCUSSION

The single discovery of general anaesthetic agents into clinical practice facilitated the development of modern surgery and helped in the expansion of the speciality of anaesthesiology. These techniques evolved first with inhalation anaesthesia, followed by local and regional anaesthesia and finally intravenous anaesthesia. Induction during general anaesthesia could be achieved by using intravenous or inhalation agents. Modern-day intravenous anaesthetics have replaced inhalation agents but none of the agents available at present meets all these requirements.^5^

Propofol is a commonly used intravenous anaesthetic and is often the induction agent of choice. However, pain on injection is a major drawback due to its highly osmotic lipid solvent and propofol's irritant effect on the vascular intima.^6^ The pain probably results from either direct irritant effects or indirect effects through activation of the plasma kinin cascade. The peripheral veins are innervated with polymodal nociceptors that mediate responses to the injection that causes pain. Other possible explanation for pain includes osmolality differences and unphysiological pH.^1^

Several physical and pharmacological strategies have been used to reduce this pain but the best means has yet to be determined.^7^ Some of them include varying the speed of injection, cooling or diluting the propofol solution, injecting propofol into large antecubital veins, pretreatments or simultaneous injection of different drugs. Drugs commonly used are Lidocaine, Tramadol, Ketamine, Sodium Thiopental, Nitroglycerin, Ketorolac, Paracetamol, NSAIDS, Metoclopramide, Ondansetron, Granisetron, Fentanyl, Remifentanil, Pethidine and Alfentanil.^8,9^

Of various methods, lidocaine pretreatment with venous occlusion or injection in the large vein is most effective. Lidocaine is a commonly used amide local anaesthetic.^10^ The efficacy profile of lidocaine as a local anaesthetic is characterized by a rapid onset of action and intermediate duration of efficacy. Reduction of pain induced by propofol injection by lidocaine may be due to local anaesthetic effect and stabilizing the kinin cascade.^11^ The observation that pain was reduced or absent when the drug was injected into an antecubital vein led to the speculation that increasing the diameter of the vein by vasodilation might reduce the incidence of pain when propofol is injected into the dorsum of the hand.^12^

This study was conducted to observe the prevalence of pain during the injection of propofol after the administration of pre-anaesthetic drug such as lidocaine. Various grading of pain was observed during propofol injection in our study. In patients pretreated with lidocaine, no pain 56 (87.5.%), mild pain 8 (12.5%) and moderate pain 0 (0%) were observed. The findings of our study also showed lidocaine were effective in the reduction of propofol-induced pain in accordance to the findings of other studies.^13,14^

A systematic review by Jalota, et al. concluded the two most efficacious interventions to reduce pain on injection of propofol were use of the antecubital vein, or pretreatment using lidocaine in conjunction with venous occlusion when the hand vein was chosen.^15^

## CONCLUSIONS

From the findings of this study, it can be concluded that the grade of pain during the injection of propofol was reduced in more than three-fourth of the patients after the administration of pre-anaesthetic drug-like lidocaine. Thus, lidocaine can be safely administered in clinical practices.
